# Sex-related differences in genetically determined Alzheimer’s disease

**DOI:** 10.3389/fnagi.2025.1522434

**Published:** 2025-03-04

**Authors:** Laura Del Hoyo Soriano, Olivia Wagemann, Alexandre Bejanin, Johannes Levin, Juan Fortea

**Affiliations:** ^1^Sant Pau Memory Unit, Department of Neurology, Hospital de la Santa Creu i Sant Pau, Biomedical Research Institute Sant Pau, Barcelona, Spain; ^2^Center of Biomedical Investigation Network for Neurodegenerative Diseases (CIBERNED), Madrid, Spain; ^3^Department of Neurology, Ludwig-Maximilians-Universität (LMU) München, Munich, Germany; ^4^German Center for Neurodegenerative Disease (DZNE), Munich, Germany; ^5^Munich Cluster for Systems Neurology (SyNergy), Munich, Germany; ^6^Barcelona Down Medical Center, Fundació Catalana de Síndrome de Down, Barcelona, Spain

**Keywords:** genetically determined Alzheimer’s disease, autosomal dominant Alzheimer’s disease (ADAD), down syndrome-associated Alzheimer’s disease (DS-AD), apolipoprotein E epsilon 4 (*APOE4*) homozygosity, sex differences in Alzheimer’s disease, disease penetrance in Alzheimer’s disease, symptom onset in Alzheimer’s disease, AT(N) biomarker trajectories in Alzheimer’s disease (amyloid, tau and neurodegeneration biomarkers)

## Abstract

We reviewed the literature on sex differences in genetically determined Alzheimer’s disease (AD), focusing on autosomal dominant AD (ADAD), Down syndrome-associated AD (DSAD), and *APOE4* homozygosity, particularly regarding disease penetrance, symptom onset and clinical progression, and trajectories for markers of amyloidosis (A), tau pathology (T) and neurodegeneration (N). Data suggests that sex differences in disease penetrance, symptom onset, and AT(N) biomarker trajectories are typically subtle for genetically determined AD populations. Noteworthy exceptions, such as increased neurodegeneration in later stages of the disease in females while similar cognitive outcomes, suggest a potential differential cognitive reserve that warrants further investigation. Additionally, the interaction between *APOE* genotype and sex reveals complex and multifaceted effects in DSAD, with potential implications for ADAD that remain underexplored. The smaller sex differences observed compared to sporadic AD offer insights into the different underlying disease mechanisms in genetically determined AD populations. Future research should prioritize sex-specific investigations in genetically determined AD, focusing on refining methodologies. This includes prioritizing longitudinal designs, adjustment for key confounders, and adherence to sex-specific guidelines.

## 1 Introduction

Alzheimer’s disease (AD) is the most common cause of dementia worldwide (Gustavsson et al., 2023), and its prevalence reveals notable differences between sexes (Alzheimer’s Disease Facts and Figures, 2024). Females account for about two-thirds of AD cases globally (Huque et al., 2023; Zhu et al., 2021), a disparity often attributed to their longer lifespans (Mielke et al., 2014). Selective survival may also play a role, as males who reach older ages often represent a biologically and genetically advantaged subset, potentially reducing their observed AD risk compared to females (Shaw et al., 2021). In addition, emerging research suggest that factors beyond life expectancy and selective survival may also play a role in shaping sex-specific risks for AD (Chêne et al., 2015; Shaw et al., 2021). For instance, hormonal changes, particularly the rapid decline in estrogen during menopause, may increase AD vulnerability in females by impairing glucose metabolism, increasing amyloid-beta deposition, and exacerbating neuroinflammation (Silva et al., 2024). Cardiovascular health disparities (Ferretti et al., 2018) and immune differences further contribute to sex-specific AD risks (Deming et al., 2018).

Research indicates not only a higher prevalence of AD in females but also a faster progression from mild cognitive impairment (MCI) to AD dementia compared to males, with females eventually losing their verbal memory advantage as the disease advances (Arenaza-Urquijo et al., 2024; Castro-Aldrete et al., 2023; Lin et al., 2015; Sohn et al., 2018). Sexual dimorphism in cognitive skills—where males typically excel in visuospatial tasks, reaction times, and mathematical reasoning, while females outperform in verbal abilities, executive functioning, attention, and episodic memory (Levine et al., 2016; Lauer et al., 2019; Sang et al., 2024; Hirnstein et al., 2023; DeCasien et al., 2022) —is particularly relevant in this context. Current cognitive measures designed to detect AD-related decline may not fully account for these sex-specific differences, potentially leading to biased diagnoses and assessments. Specifically, the female verbal memory advantage may mask early cognitive deficits, delaying AD diagnosis when standard verbal-based memory tests are used. This could contribute to the perception of faster cognitive decline in later stages for females, as deficits become more apparent when the verbal advantage diminishes.

Sexual dimorphism also extends to brain structure. Males generally have larger overall brain sizes, including absolute gray and white matter volumes, while females exhibit proportionally larger gray and white matter volumes and thicker cortices when adjusted for total brain volume (TBV) or intracranial volume (ICV) (Eliot et al., 2021). Females also show relatively larger hippocampal volumes—an area critical for memory and AD pathology—while males display larger amygdala and putamen volumes when normalized for TBV or ICV (Eliot et al., 2021). Although these structural and functional differences often show small and inconsistent effects across studies, they underscore the importance of considering sex as a critical factor in understanding AD. Thus, sexual dimorphism across various levels—including genetics, hormones, brain structure, and cognition—along with cultural influences (Reilly, 2012; Springer et al., 2012) likely contributes to sex-related disparities in AD prevalence.

Given the potential role of sexual dimorphism in AD pathology, understanding sex-specific trajectories in AD biomarkers has become a critical focus in research. Efforts to elucidate these differences often rely on the AT(N) biomarker framework, which characterizes AD pathology through amyloid plaques (A), tau tangles (T), and neurodegeneration (N). Amyloid-beta (A) biomarkers, such as CSF amyloid-*β* (Aβ)42 levels, Aβ42/Aβ40 ratios, and amyloid-PET scans, consistently show no significant sex differences (Mielke, 2020). Conversely, tau biomarkers (T) present a divergent picture; while plasma and CSF phosphorylated tau (p-tau181) levels are comparable between sexes, several studies have shown a more pronounced tau accumulation in tau-PET scans in key brain regions in females, even after adjusting for overall disease severity (Buckley et al., 2019). This observation is supported by autopsy studies indicating higher neurofibrillary tangle densities in females (Liesinger et al., 2018). Regarding neurodegeneration (N), some studies suggest males have higher concentrations of neurofilament light chain (NfL) in CSF (Bridel et al., 2019), a marker of axonal damage, but other studies have not found differences in plasma. Additionally, MRI biomarkers present mixed results across sexes (Ferretti et al., 2018). Importantly, a recent study adds a layer of complexity in the interpretation of sex differences in AD biomarkers: While plasma p-tau181 levels were found to be similar between sexes, females showed greater neurodegeneration, faster cognitive decline, and a higher risk of developing AD dementia associated with elevated p-tau181 compared to males (Tsiknia et al., 2022). These findings suggest that sex differences may affect the clinical interpretation of plasma p-tau181 as an AD biomarker, paralleling previously reported challenges in assessing cognitive changes related to verbal memory.

In addition, recent multi-omics studies have provided valuable insights into the molecular mechanisms underlying sex-differences in AD (Guo et al., 2021). Transcriptomic analyses link gene downregulation in female neurons and transcriptional activation in male oligodendrocytes to disease progression (Mathys et al., 2019). Females show more immune-related gene expression changes, while males exhibit differences in synaptic signaling and autophagy (Paranjpe et al., 2021). Females also show greater immune and neuronal pathway alterations, with stronger associations to amyloid and tau pathologies (Deming et al., 2018), and metabolomic findings indicate dysregulated lipid metabolism and energy pathways, particularly in female *APOE4* carriers (Shang et al., 2020) with distinct methylation and RNA profiles (Cao et al., 2019; Mano et al., 2017). The *APOE* ε4 allele is the strongest genetic risk factor for late-onset AD. Research has consistently shown a more pronounced impact on females, who show worse memory performance, global cognition, and higher cerebrospinal tau levels compared to males (Walters et al., 2023; Yan et al., 2021).

Genetically determined AD offers unique opportunities to understanding sex effects on AD pathophysiology. Late onset sporadic AD dementia cases, even when confirmed by biomarkers, often involves additional co-pathologies, complicating the disease landscape. At autopsy, AD pathology is frequently accompanied by other neurodegenerative conditions such as vascular dementia, Lewy body dementia, or other forms of pathology. These additional conditions, for which biomarkers are less robust, make it more challenging to disentangle and analyze sex-related differences in sporadic AD (Mortimer, 2013). This complex landscape can significantly influence the observed differences between sexes in late-onset sporadic AD adding further complexity to the study of sex-related differences in this population. In contrast, genetic forms of AD might offer a clearer perspective on disease development and sex-related variations, as co-pathologies are less frequent in these cases. However, it is important to note that Cerebral Amyloid Angiopathy (CAA) is more prevalent and severe in these genetic forms than in sporadic AD (Carmona-Iragui et al., 2019). Three genetic forms of AD have been proposed; autosomal dominant AD (ADAD) (Bateman et al., 2012), Down syndrome associated AD (DSAD) (Fortea et al., 2020, 2021), and more recently, apolipoprotein E epsilon 4 (*APOE4*) homozygosity (Fortea et al., 2024).

In ADAD, mutations in the *amyloid precursor protein* (*APP*), *presenilin 1* (*PSEN-1*), or *presenilin 2* (*PSEN-2*) gene lead to altered processing of the amyloid precursor protein, resulting in an increased ratio of longer amyloid-*β* (Aβ) fragments, particularly Aβ42. These longer Aβ fragments are more prone to aggregation and are associated with early and extensive deposition of amyloid plaques in the brain. This amyloid deposition triggers a cascade of neurodegenerative processes that lead to neuronal death the early onset of clinical symptoms (i.e., before 65 years of age), and eventual dementia. Mutations in *APP*, *PSEN1*, and *PSEN2* have an estimated prevalence of 5.3 per 100,000 persons (i.e., 5.3e-05% worldwide) (Lanoiselée et al., 2017). Among these, *PSEN1* mutations are the most common, followed by *APP* and *PSEN2* mutations.

DS is the most frequent genetic cause of intellectual disability (ID), affecting approximately 0.14% of the general population. Due to the triplication of the *APP* gene, which is encoded on chromosome 21, individuals have near full penetrance of AD dementia (Fortea et al., 2021) and AD now represents the main cause of death in adults of this population (Iulita et al., 2022). Notably, ADAD and DSAD have been recognized as genetic forms in the new Alzheimer’s Association criteria (Dubois et al., 2014).

We have recently proposed *APOE4* homozygotes as another form of genetic AD as it also fulfills the key features of genetically determined AD: (1) near-full penetrance of the disease (defined biologically), (2) predictability of the age at symptom onset and of clinical changes, and (3) a consistent sequence of biomarker and pathological alterations. The three forms of the disease show striking similarities in these features (Bateman et al., 2012; Fortea et al., 2020, 2021, 2024), suggesting that *APOE4* homozygotes could be reclassified as a distinct form of genetically determined AD. Notably, approximately 2% of the global population is homozygous for *APOE4*, accounting for 15–20% of AD cases.

Our review will examine sex differences in three genetically determined AD populations: ADAD, DSAD, and *APOE*4 homozygotes. For each, we will summarize studies on sex differences in biomarkers, cognition, and neural dimorphism unrelated to AD that may influence disease risk and progression. We will then explore sex effects on disease penetrance, symptom onset, clinical progression, and AT(N) biomarker changes, including interactions between sex and *APOE* status in ADAD and DSAD. To deepen understanding, we will review multi-omics studies to provide a comprehensive overview of sex effects in genetically determined AD.

## 2 Sex-related differences in ADAD

To our knowledge, six studies have investigated sex-related differences in ADAD outcomes, with five of them being cross-sectional. Three studies were conducted as part of *The Colombian Alzheimer’s Prevention Initiative (API) Registry* (Vila-Castelar et al., 2020, 2022, 2023), focusing on members of the PSEN1 E280A family from Antioquia, Colombia. Another Latin-American cohort from Jalisco, Mexico, involving *PSEN1* mutation carriers, has published few studies on the clinical phenotype and progression of AD, some of which included sex-related analyses (Dumois-Petersen et al., 2020). The final two studies investigating sex-related differences come from the international Dominantly Inherited Alzheimer Network (DIAN) cohort (Kommaddi et al., 2023; Wagemann et al., 2024) which recruited family members with *APP, PSEN1*, and *PSEN2* mutations from Asia, Australia, Europe, and the Americas. Notably, only one of these studies included longitudinal data (Kommaddi et al., 2023).

Regarding sexual dimorphism, female asymptomatic mutation carriers exhibit significantly higher episodic verbal memory scores compared to their male counterparts, a trend observed in both the Colombian cohort and in DIAN (Vila-Castelar et al., 2022; Wagemann et al., 2024). Interestingly, this sex difference is evident since childhood (Fox-Fuller et al., 2021). The study of 1,354 children aged 6 to 16 years from the Colombian cohort, including 265 with the *PSEN1* variant, showed that girls outperformed boys in working memory, perceptual reasoning, and verbal comprehension, regardless of genetic status. The disparity in working memory, however, was especially pronounced in the *PSEN1* carriers. No differences in other cognitive outcome shave been explored. In addition, the DIAN cohort which included 436 participants (257 mutation carriers and 179 non-carriers) of approximately 7 years before expected symptom onset, revealed that there was a Brain Age Gap (calculated by predicting the brain’s “age” based on imaging features such as cortical thickness, gray matter volume, and white matter integrity, and then subtracting the individual’s chronological age) of approximately 3 years greater in males than females regardless of mutations status (Millar et al., 2023). Such findings underscore that nuanced differences in cognitive resilience and brain aging trajectories may exist in this population. No further sex differences have been found in other brain structures [e.g., hippocampus and amygdala (Vila-Castelar et al., 2022; Wagemann et al., 2024)] or in cortical thickness (Wagemann et al., 2024) in pre-symptomatic ADAD.

Regarding sex effects on AD penetrance, both the Colombian (Aguirre-Acevedo et al., 2016) and Mexican kindred (Dumois-Petersen et al., 2020) as well the cross-sectional DIAN cohort studies (Wagemann et al., 2024) demonstrated full disease penetrance without significant sex differences in symptom onset or clinical progression. While the female advantage in verbal memory generally diminishes with age, these studies indicate that this does not significantly affect the clinical onset or progression of AD. However, the longitudinal study revealed some interesting sex differences in clinical progression (Kommaddi et al., 2023). Specifically, females demonstrated slower cognitive decline in tasks involving logical verbal memory. Conversely, in tasks requiring executive function—symptomatic female mutation carriers declined faster than their male counterparts. No significant sex differences were found in other cognitive outcomes, including episodic verbal memory tasks or in the Mini-Mental State Examination (MMSE). These findings suggest that while females may show greater resilience in specific verbal memory tasks, they may experience more rapid decline in areas like executive function, which are not typically emphasized in AD-related examinations.

Regarding the sequence of AD biomarker changes, most studies do not show significant sex differences in amyloid (CSF Aβ42/40 ratio), tau (CSF p-tau181, t-tau levels), or markers of neurodegeneration, with two exceptions: A study from the Colombian Kindred found that female carriers exhibited a greater increase in plasma NfL levels compared to male carriers, but no significant differences in p-tau217 levels. Interestingly, despite the increased plasma NfL levels in females, this did not correlate with differences in cognitive performance (Vila-Castelar et al., 2023). Furthermore, the cross-sectional study from the DIAN cohort noted that with disease progression, symptomatic female carriers showed more increased cortical thinning and decreased volumes of the hippocampus and amygdala compared to male carriers, highlighting a greater degree of neurodegeneration in advanced stages for females (Wagemann et al., 2024). These findings suggest that while core biomarker changes are mostly similar between sexes, distinct differences in neurodegeneration and cognitive resilience may become evident, particularly in the later stages of the disease.

The role of *APOE* in ADAD, particularly among *PSEN1* mutation carriers, has shown mixed findings. Several studies in the Colombian kindred suggested that carriers of the *APOE* ε4 allele developed earlier dementia onset compared to non-carriers (Pastor et al., 2003; Langella et al., 2023). In contrast, a study conducted in the Mexican kindred found the opposite effect, suggesting a later onset for *APOE* ε4 carriers (Valdez-Gaxiola et al., 2023). Adding to the complexity, other studies in the same cohort (Langella et al., 2024; Vélez et al., 2016) found no significant effect of *APOE4* on symptom onset and trajectories, while noted that the *APOE* ε2 allele delayed the onset of clinical symptoms by approximately 8 years. Similarly, the *APOE3* Christchurch variant has been associated with delayed cognitive impairment in *PSEN1* carriers (Quiroz et al., 2024). Broader studies involving various ADAD families have indicated differences between APP and *PSEN1* mutations, which may obscure the effects of *APOE* in pooled analyses (Lanoiselée et al., 2017). Of note, the role of *APOE4* in ADAD has shown to be variable across the lifespan and it is possible that *APOE4* may provide some biological or cognitive benefit in younger *PSEN1* carriers. Regarding biomarker progression, a recent study in the Colombian kindred found that *APOE4* accelerates age-related plasma NfL increases and *APOE* ε2 attenuates the relationship between higher plasma NfL levels and cognitive decline in ADAD (Langella et al., 2024).

Regarding the interaction between *APOE* ε4 and sex, studies in the Colombian cohort found no significant relationship between *APOE*, memory, amyloid burden, or cerebral hypometabolism, though the sample size was small, with only 18 *APOE4* carriers per sex (Vila-Castelar et al., 2022). The DIAN study reported no significant differences between sexes for *APOE4* status but did not examine the interactive effects between *APOE4* status and sex on AD outcomes. However, a 2014 metanalysis (Ryman et al., 2014) that examined combined dataset—including the DIAN database and two large kindreds of Colombian (PSEN1 E280A) and Volga German (PSEN2 N141I) ancestry—found no interaction between sex and *APOE*4 status on symptom onset or disease progression. Neither sex nor *APOE4* status independently influenced AD outcomes. Further research is required to validate the absence of interactive effects in this population.

Finally, only two studies from the DIAN cohort have investigated CSF proteomics in ADAD. The first reported no significant impact of sex or *APOE*4 status on proteomic changes or their progression relative to estimated years to disease onset (Johnson et al., 2023). Similarly, the second study compared proteomic profiles between mutation carriers and controls, with no explicit sex-related differences observed in CSF proteomics or associated biomarker pathways (Van Der Ende et al., 2023).

In conclusion, current research on sex-related differences in ADAD indicates that female asymptomatic mutation carriers consistently demonstrate higher verbal memory scores from childhood, highlighting the influence of sexual dimorphism rather than a specific sex effect on AD progression. Despite this verbal advantage, most studies have found no significant sex differences in AD penetrance, symptom onset and progression, or biomarker changes. However, in advanced disease stages, females may exhibit more pronounced brain atrophy, particularly in regions such as the hippocampus and amygdala, although this does not consistently correlate with greater cognitive decline. See Table 1 for an overview of the published studies investigating the role of sex in ADAD.

**Table 1:** Overview of studies investigating the role of sex in genetically determined Alzheimer’s disease.

Condition	Study/Reference	Sexual dimorphism findings	AD-Related Findings
ADAD	Vila-Castelar et al. (2020)	Female carriers have higher episodic verbal memory scores pre-symptomatically.	No sex differences in hippocampal or other brain volume measures.
	Vila-Castelar et al. (2022)	Females outperform males in working memory, perceptual reasoning, and verbal comprehension. No sex differences in other cognitive domains.	
	Vila-Castelar et al. (2023)		Plasma NfL levels increased more in females; no sex differences in amyloid or tau biomarkers.
	Dumois-Petersen et al. (2020)		No sex differences in symptom onset or progression.
	Wagemann et al. (2024)		No sex differences in amyloid or tau PET; females show greater cortical thinning and hippocampal atrophy in symptomatic stages.
	Kommaddi et al. (2023)		Females have slower cognitive decline in verbal tasks but faster decline in executive function.
	Millar et al. (2023)	Males show advanced structural brain aging, with their predicted brain age exceeding their chronological age; no differences in memory and executive function.	
	Ryman et al. (2014)		No interaction between sex and *APOE4* status on symptom onset or progression.
	Johnson et al. (2023)		No significant sex-related differences in CSF proteomic changes or their progression across years to estimated disease onset.
	Van Der Ende et al. (2023)		No explicit sex-related differences in CSF proteomics or biomarker pathways.
DSAD	Iulita et al. (2023)	No sex differences in hippocampal volume normalized by ICV, or episodic memory.	No sex differences in penetrance, symptom onset, or clinical progression. *APOE4* carrier females diagnosed ~3 years earlier than noncarriers, with poorer episodic memory; no significant differences in males.
	Startin et al. (2020)	Females outperform males in receptive language abilities across the lifespan. No differences in overall general cognitive function.	
	del Hoyo Soriano et al. (2018)	Females outperform males in expressive structural language. No sex differences in general cognitive function or social cognition.	
	Larsen et al. (2024)		Female *APOE4* carriers exhibit earlier symptom onset than male counterparts.
	Lai et al. (1999)		Female *APOE4* carriers have a higher risk of developing AD compared to their male counterparts.
	Lai et al. (2020)		No significant sex differences in penetrance, symptom onset, or progression.
	Martá-Ariza et al. (2025)	Consistent inflammatory upregulation in females.	No differences in tau deposition patterns.
*APOE44*	Fortea et al. (2024)	Females have smaller adjusted hippocampal volumes than males.	Similar disease penetrance and age at symptom onset, cognitive progression and AT(N) biomarker trajectories across sexes.
	Jansen et al. (2022)		No significant sex differences in clinical progression or biomarker trajectories.
	Zou et al. (2023)	No significant differences in global cognition, episodic memory, or executive function.	Males show earlier decreases in CSF Aβ42 levels but no differences in amyloid PET or tau biomarkers.
	Khoury et al. (2024)		Female homozygotes have worse neuropsychiatric symptoms and reduced cortical thickness.
	Yan et al. (2021)		Females exhibit greater tau-related susceptibility; males require two *APOE4* copies to reach tau levels observed in females with one copy.

Table summarizes studies investigating the role of sex in genetically determined forms of Alzheimer’s disease (AD), specifically autosomal dominant AD (ADAD), Down syndrome-associated AD (DSAD), and *APOE*4 homozygosity. Findings are organized into two categories: (1) Sexual Dimorphism Findings, which pertain to inherent sex-based differences in cognitive, structural, or molecular characteristics independent from AD; and (2) AD-Related Findings, which include sex-related investigations in AD-specific outcomes such as disease penetrance, symptom onset, clinical progression, and biomarker trajectories. Abbreviations: *APOE*4 (Apolipoprotein E epsilon 4), NfL (Neurofilament Light), ICV (Intracranial Volume), PET (Positron Emission Tomography).

## 3 Sex-related differences in DSAD

Compared to ADAD, there are more studies investigating the sex-differences in DSAD outcomes (Iulita et al., 2023; Lai et al., 2020; Larsen et al., 2024; Mhatre et al., 2021; Rubenstein et al., 2024; Rubenstein et al., 2020). However, only one has also looked at the sequence of AT(N) biomarker changes across age and sex in 628 adults with DS from the Down Alzheimer Barcelona Neuroimaging Initiative (DABNI), Spain and the University of Cambridge, UK (Iulita et al., 2023).

Regarding sexual dimorphism, a recent study on cognitive abilities in DS (Startin et al., 2020) examined 602 individuals aged 3 months to 73 years and found that males consistently scored lower than females in receptive language abilities across the lifespan. Similarly, another study reported that during adolescence, females outperformed males in expressive structural language using speech samples (del Hoyo Soriano et al., 2018). Additionally, research on asymptomatic young adults with DS targeting multiple cognitive domains, found that females excelled males only in episodic memory and executive functioning (de Sola et al., 2015). Regarding functional and structural brain differences, DS male brains are generally larger than female brains, with greater cortical thickness (Romano et al., 2015) and larger unadjusted hippocampal volumes (Romano et al., 2015). However, most studies show no significant sex-related differences in adjusted hippocampal volumes or glucose metabolism for asymptomatic adults with DS (Iulita et al., 2023; Koenig et al., 2021). Nonetheless, a study–based on 54 MRI scans- found smaller hippocampal volumes in asymptomatic females (controlling for intracranial volume) (Peven et al., 2022).

Mixed results have been found regarding disease penetrance across sexes in the DS population, possibly due to methodological differences between studies. Some studies report a greater risk of dementia in males for older age ranges (Mhatre et al., 2021; Schupf et al., 1998), while others find increased risk in females aged 40 to 54 but no sex differences in those younger than 40 or older than 55 (Rubenstein et al., 2020). Some research shows a mildly greater risk for females across all ages (Lai et al., 1999), while other studies find no sex differences at any age range (Iulita et al., 2023; Lai et al., 2020; Rubenstein et al., 2024). Of such, a recent epidemiological study in adults with DS enrolled in Medicaid or Medicare between 2011 and 2019 (*N* = 132,720) identified via ICD codes, found no sex-related differences in prevalence and incidence of AD by age (Rubenstein et al., 2024). The inconsistencies across studies may be attributed to variations in study design, such as longitudinal vs. cross-sectional, prospective vs. retrospective studies and research cohorts vs. epidemiological studies based medical records or healthcare reimbursement requests. The retrospective and epidemiological approaches may be subject to biases such as referral differences, coding errors, variations in provider documentation, or disparities in healthcare access and utilization. Additionally, age ranges in the studies varied widely (20 to 70 years), and sample sizes ranged from as few as 21 to over 100,000 participants. Overall, larger and more recent prospective studies, such as those from the DABNI cohort and the large epidemiological study in adults with DS enrolled in Medicaid or Medicare in the U.S have not found significant sex differences in disease risk in the DS population (Iulita et al., 2023; Rubenstein et al., 2024). These findings suggest that, despite earlier conflicting reports, there appears to be no substantial sex difference in AD penetrance among individuals with DS.

Regarding sex-differences in symptom onset, studies have shown mixed results as well. In the 1990s, some studies indicated an earlier onset in males (Schupf et al., 1998), while others found this trend in females (Lai et al., 1999). However, studies from 2020 onwards have found no significant differences in symptom onset across sexes (Iulita et al., 2023; Lai et al., 2020). Regarding clinical progression, longitudinal research shows that rate of cognitive decline does not differ by sex when transitioning from the preclinical stage to prodromal AD (Hartley et al., 2020). Additionally, a recent a meta-analysis with the assessment of mortality data from US death certificates (*n* = 77,347 case records [49%] female) and from a longitudinal cohort study (DANBI, *n* = 889 individuals; 46% female; 3.2 [2.1] years of follow-up) found no sex differences in the age of death among the older participants, for whom AD is the predominant cause of mortality (Iulita et al., 2022). The large epidemiological study involving adults with DS enrolled in Medicaid or Medicare in the U.S. found no differences in the incidence of AD dementia between sexes (Rubenstein et al., 2024; Rubenstein et al., 2020).

Regarding the sequence of biomarkers by sex, the study conducted on the DABNI and UK cohorts (Iulita et al., 2023) found that males and females exhibit similar trajectories with age for markers of amyloidosis (CSF amyloid-*β* 42/40 and amyloid-PET), tau pathology (CSF and plasma phosphorylated-tau181), and neurodegeneration (CSF and plasma neurofilament light, total-tau, fluorodeoxyglucose-PET, and MRI). However, it is important to note that tau-PET data was not available in this study, leaving a gap in the assessment of sex differences in tau deposition. Overall, the findings suggest that there is no significant sex effect on the trajectory of AD biomarkers in the DS population, though further research is needed to confirm these results, particularly with the inclusion of tau-PET data.

Several studies show that DS adults carrying the *APOE* ε4 allele face a higher risk (Prasher et al., 2008) of developing AD dementia and tend to have an earlier age of onset (Bejanin et al., 2021; Silverman et al., 2013) and a higher degree of cognitive decline (Gorijala et al., 2024) and earlier changes in amyloid (cerebrospinal fluid A*β*1-42/1-40 and amyloid positron emission tomography), tau (plasma phosphorylated tau 181), and neurodegeneration (cerebral glucose hypometabolism and hippocampal atrophy) biomarkers (Bejanin et al., 2021). In addition, *APOE* ε2 carriers have shown an increased protection against AD pathology (Lai et al., 1999). These trends are in line with observations in sporadic AD, though they tend to be less pronounced in DSAD. Regarding the interaction between *APOE* and sex on DSAD outcomes, conflicting results have been found. Some studies have reported that females ε4 carriers tend to be diagnosed at an earlier age than their male counterparts (Larsen et al., 2024), while others have found no such association (Iulita et al., 2023; Lai et al., 1999). However, the study in the Spanish and British (Iulita et al., 2023) cohort found that females with an *APOE* ε4 allele had poorer episodic verbal memory and were diagnosed with Alzheimer’s disease an average of 3 years earlier than non-carriers, while no such differences were observed between male ε4 carriers and non-carriers. At the biomarker level, female ε4 carriers showed a lower CSF Aβ42/Aβ40 ratio and reduced hippocampal volume compared to non-carriers, a pattern not seen in males. Findings from this study underscore the complexity of the interaction between sex and *APOE* in DSAD, highlighting the need for further research to clarify these relationships and their implications for clinical outcomes.

Significant transcriptomic sex differences between DSAD and sporadic AD have been identified using spatial and single-nucleus transcriptomics (Miyoshi et al., 2024). This recent study revealed that the role of sex differed between DSAD and sporadic AD. In DSAD, transcriptomic differences between sexes were more pronounced, with females showing consistent upregulation of inflammatory and glial genes across brain regions and stronger neuroinflammatory signatures, particularly in late stages of the disease. This contrasts with sporadic AD, where sex differences were more region- and stage-specific. Another recent study (Martá-Ariza et al., 2025) evaluated the Aβ plaque proteome in four cohorts—ADAD, late-onset AD, DSAD, and controls—finding significantly increased amyloid-β and tau pathologies in DS, but no significant sex effects on proteomic profiles. However, this study did not examine the relationship between sex and proteomic profiles specific to each AD population. Similarly, a study of plasma metabolomic profiles in individuals with DS (ages 10–63) found no significant effects of age or sex on metabolite concentrations or patterns, nor any sex-specific differences within age groups (Antonaros et al., 2020).

In conclusion, females with DS exhibit higher verbal scores from childhood, and smaller hippocampal areas, mirroring the sexual dimorphism observed in the general population. When examining AD outcomes, recent prospective studies with larger samples have found no significant sex differences in disease penetrance, symptom onset and clinical progression and biomarker trajectories. However, females with DS and the *APOE4* allele tend to be diagnosed earlier and exhibit lower CSF Aβ42/Aβ40 ratios and reduced hippocampal volumes than males’ counterparts, indicating an interaction between sex and *APOE4* in the DS population. Furthermore, multi-omics findings reveal pronounced transcriptomic sex differences in DSAD compared to sporadic AD, with DSAD females showing consistent upregulation of inflammatory and glial genes, particularly in late stages of the disease. See Table 1 for an overview of the published studies investigating the role of sex in DSAD.

## 4 Sex-related differences in *APOE4* homozygotes

Most studies tend to group *APOE4* heterozygotes and homozygotes into a single ‘*APOE4* carriers’ category, largely due to sample size constraints. However, there is still a body research focused on examining the distinct effects of *APOE4* dosage, particularly the differences between homozygosity heterozygosity, and noncarriers on penetrance, clinical progression, and biomarker changes by sex (Bocancea et al., 2023). Although this section will primarily address sex differences in *APOE4* homozygotes as a genetically determined form of AD, we will also discuss literature on the broader implications of *APOE4* dosage effects at the end of this section.

Due to sample size limitations, few studies have specifically analyzed sex differences in AD penetrance, symptom onset, clinical progression, and the biomarker sequence in *APOE4* homozygotes (Fortea et al., 2024; Jansen et al., 2022; Zou et al., 2023). Among the research available, the two most extensive studies to date are cross-sectional. One study analyzed data from 3,297 brain donors from the National Alzheimer’s Coordinating Center (NACC) and10,039 individuals from 5 large prospective cohorts (Fortea et al., 2024). The second study utilized data from the 85 cohorts of the Amyloid Biomarker Study (Jansen et al., 2015), an ongoing global data-pooling initiative that began in 2013, and conducted a pooled analysis on 19,097 participants, including 783 *APOE4* homozygotes (Jansen et al., 2022).

Regarding sexual dimorphism among individuals with *APOE4* homozygosity, studies have shown that asymptomatic males exhibit worse global cognition, episodic memory, executive and visuospatial function compared to females, while similar attentional levels and expressive language outcomes (Zou et al., 2023). Additionally, adult females with *APOE*4 homozygosity have significantly smaller hippocampal volumes (adjusted for ICV) than their male counterparts across all age ranges and AD statuses (Fortea et al., 2024).

In terms of disease penetrance, research consistently shows that males and females with *APOE4* homozygosity have a similar risk and predictability of the age at AD diagnosis (Fortea et al., 2024; Jansen et al., 2022; Therriault et al., 2021; Zou et al., 2023). Importantly, despite smaller adjusted hippocampal volumes in females (Fortea et al., 2024), this did not lead to greater cognitive decline over time. The sequence of biomarker changes with age in *APOE*4 homozygotes is strikingly similar to that described in ADAD and DSAD and largely sex-independent. Among the biomarkers examined, one of the few significant findings in one of the cross-sectional studies was an earlier decrease in Aβ42 levels in males (Fortea et al., 2024), which should be interpreted cautiously due to the limited number of younger participants. Additionally, amyloid PET scans did not reveal any sex differences, suggesting that the Aβ42 trend may not reflect broader amyloid pathology differences between sexes. Finally, a recent study (Khoury et al., 2024) involving 752 patients with an AD diagnosis reported that female *APOE4* homozygote carriers had worse neuropsychiatric symptoms and reduced cortical thickness, particularly in the medial-lateral temporal regions, compared to *APOE4* homozygous males. However, these findings are based on cross-sectional data in symptomatic patients only, which limits the ability to determine quantify reductions or increases in cortical thickness as true changes in gray matter related to AD pathology.

To conclude, research in *APOE4* homozygotes targeting sex-related differences indicates that asymptomatic females have better cognitive performance than their male counterparts but exhibit smaller hippocampal volumes across all age ranges possibly reflecting the sexual dimorphism rather than AD risk. Indeed, regarding AD outcomes, no significant sex differences in disease penetrance, symptom onset, progression, or biomarker trajectories for *APOE4* homozygotes, suggesting similar risks for males and females in this subgroup.

A substantial body of research explores the effects of *APOE4* dosage on penetrance, clinical progression, and biomarker changes by sex, with mixed findings (Belloy et al., 2023; Bocancea et al., 2023; Farrer et al., 1997; Liu et al., 2022). For instance, Bocancea et al. (2023) conducted a multicenter longitudinal study with 366 participants with MCI or dementia, including 71 *APOE4* homozygotes, and found no significant cross-sectional or longitudinal effects of *APOE4* status on cognition and cortical thickness when sex was considered. Additionally, *APOE4* status did not significantly moderate the association between tau pathology and cognitive decline or cortical thinning when stratified by sex. In contrast, another study with 117 participants with AD, including 21 *APOE4* homozygotes, found a sex-specific response to *APOE4* dosage: males required two copies of the allele to reach tau levels that females achieved with just one, suggesting higher susceptibility in females to the effects of *APOE4* on tau pathology (Yan et al., 2021). This finding aligns with earlier studies (Farrer et al., 1997) which showed that *APOE4* heterozygosity is sufficient to increase AD risk in females, while *APOE*4 homozygosity is necessary in males. The inconsistencies between Bocancea et al. (2023) and the remaining studies may be attributed to different methodologies and statistical approaches (e.g., use of linear mixed-effects models with sex and *APOE*4 status as interactive variables versus sex-stratified analyses).

Metabolomic and transcriptomic studies highlight sex-specific differences among *APOE4* carriers. Females show metabolic vulnerabilities, such as acylcarnitine C10’s association with increased CSF p-tau and higher proline levels linked to reduced brain glucose uptake (Arnold et al., 2020). Single-cell transcriptomic reveal that female *APOE*4 carriers display unique transcriptional changes in excitatory neurons and astrocytes and a distinct neutrophil phenotype characterized by IL-17/IL-1 gene expression linked to cognitive impairment (Yu et al., 2024). These molecular differences suggest heightened female susceptibility to *APOE*4-driven neuroinflammation and neurodegeneration.

To add another layer of complexity, the *APOE4-*AD association varies by race and ethnicity, with a stepwise decrease in effect estimates across East Asian, non-Hispanic White, non-Hispanic Black, and Hispanic individuals (Belloy et al., 2023). While *APOE4* dosage generally increases AD risk in women across races, the magnitude and manifestations of this effect differ by racial and ethnic background (Belloy et al., 2023; Tang et al., 1998) suggesting that findings from research focused predominantly on Caucasian populations may not fully capture sex-specific risks in diverse racial and ethnic groups. These observations highlight the intricate interplay between genetic, racial, and sex-specific factors in AD and underscore the critical need for racial and ethnic stratification in studies of *APOE4* effects to improve our understanding of these mechanisms in both men and women.

In summary, although no significant sex differences are observed in AD outcomes among *APOE4* homozygotes, the impact of *APOE* dosage on AD risk might differ between males and females. This discrepancy could be due to several factors. For example, in heterozygotes, the interaction between *APOE4* and other genes might trigger sex-specific biological mechanisms, whereas in homozygotes, the strong impact of two *APOE4* alleles could overshadow these differences. Additionally, genetic resilience and compensatory mechanisms may vary by sex in heterozygotes but may be insufficient to counterbalance the effects in homozygotes. See Table 1 for an overview of the published studies investigating the role of sex in *APOE4* homozygotes.

## 5 Concluding remarks

Genetically determined AD has been critical in our understanding of the pathophysiology of AD and offers the unique opportunity of studying the role of sex in disease penetrance, predictability of the age at symptom onset and of clinical changes, and biomarker trajectories within the AT(N) framework in these more predictable forms of the disease.

**Figure 1:**
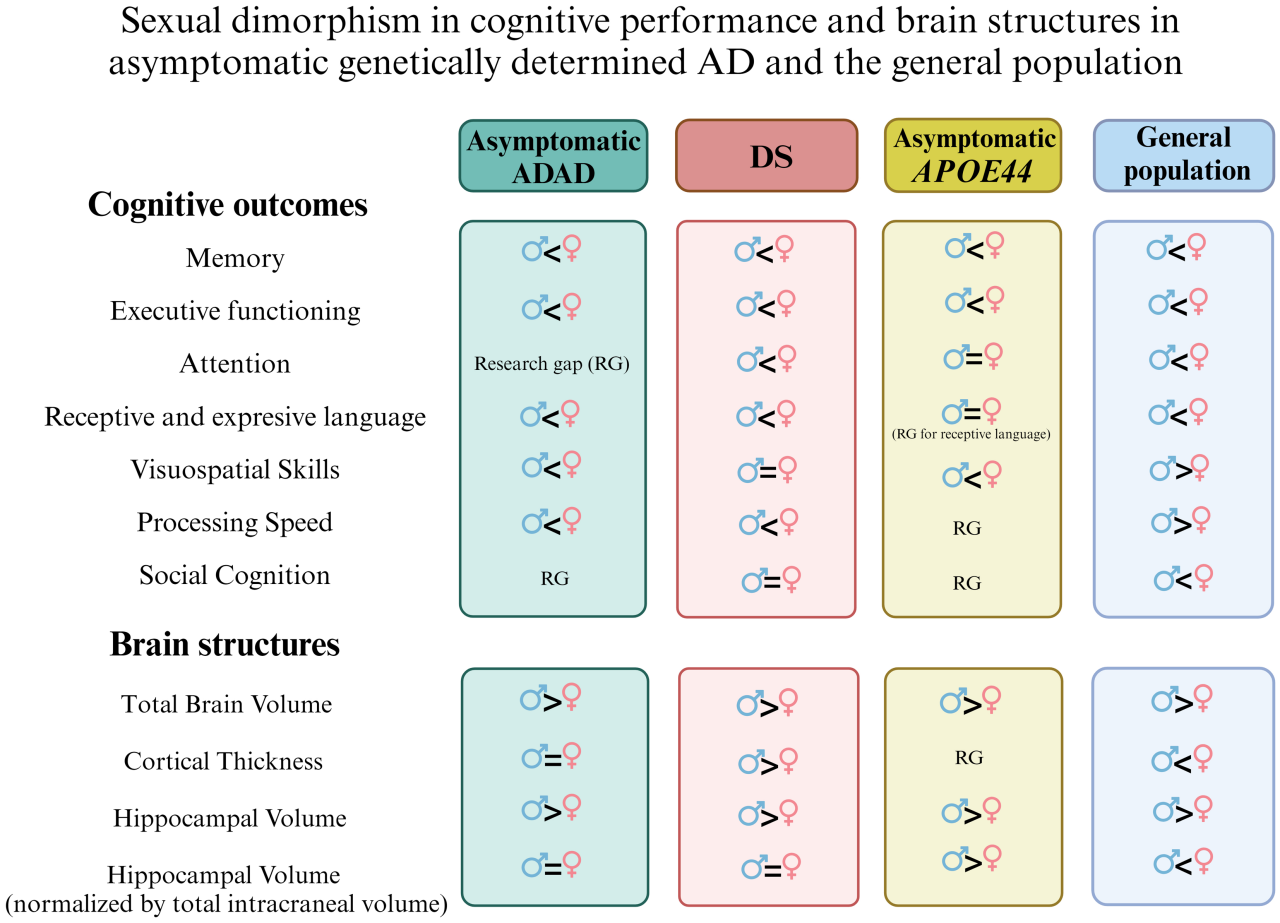
Illustration of sexual dimorphism in cognitive performance and brain structures across asymptomatic genetically determined Alzheimer’s disease (AD) populations (including Down syndrome [DS], autosomal dominant AD [ADAD], and *APOE44* carriers) compared to the general population. The figure highlights differences in total brain volume, cortical thickness, and hippocampal volume (normalized by total intracranial volume), with females generally exhibiting smaller brain structures compared to males. However, after adjustment for intracranial volume, sex differences in hippocampal volume largely disappear (except for *APOE44* and the general population). Cognitive differences between sexes are mild, with females showing an advantage in episodic memory across population. Notably, research gaps persist in understanding sex differences in some cognitive areas and brain structures.

First, there is a consistent sexual dimorphism in cognitive function across genetically determined AD, with females showing a cognitive advantage before AD onset. Structurally, females generally have smaller brain and hippocampal volumes compared to males; however, when normalized by ICV, no significant sex differences in hippocampal volume are observed in DS (with one study as an exception) or in ADAD. However, asymptomatic *APOE4* homozygous females tend to have smaller adjusted hippocampal volumes compared to their male counterparts, which contrasts with findings in the general population. Similarly, lower cortical thickness has been observed in asymptomatic DS females compared to males, again opposing trends typically seen in the general population. This difference in cortical thickness has not been observed in asymptomatic ADAD and remains unexplored in asymptomatic *APOE4* homozygotes. Significant gaps in our understanding of these differences highlight the need for further research on sexual dimorphism in cognitive and structural outcomes in these populations (Figure 1).

**Figure 2:**
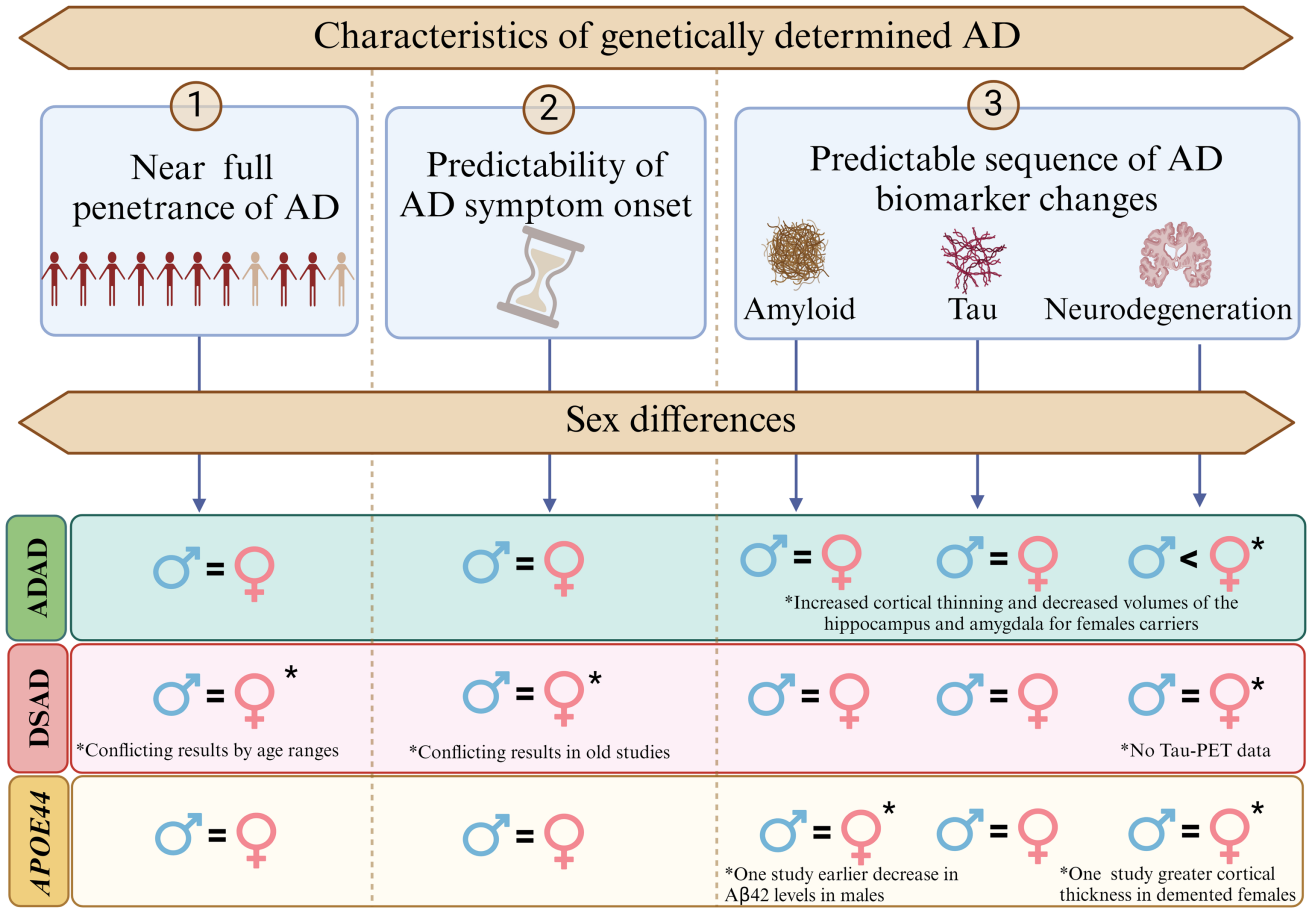
Sex differences in penetrance, symptom onset, and biomarker progression in Alzheimer’s disease (AD) across genetically determined AD populations. This figure illustrates the near-full penetrance of AD in autosomal dominant Alzheimer’s disease (ADAD), Down syndrome-associated AD (DSAD), and *APOE44* carriers, alongside the predictable sequence of AD biomarker changes (amyloid deposition, tau accumulation, and neurodegeneration). No significant sex differences were observed in most aspects of penetrance, symptom onset, or biomarker progression. However, notable exceptions include increased cortical thinning and reduced hippocampal and amygdala volumes in female ADAD carriers compared to males. Additionally, earlier decreases in Aβ42 levels were reported in *APOE44* males, while greater cortical thickness was observed in *APOE44* females. Conflicting findings were noted in DSAD for penetrance and symptom onset, with larger recent studies reporting no significant sex differences.

**Figure 3:**
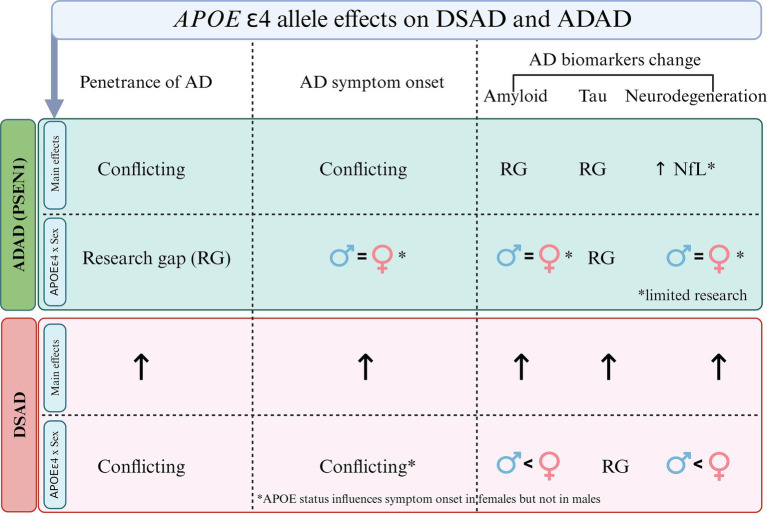
Overview of the main effects of *APOE4* and its interaction with sex in Alzheimer’s disease (AD) penetrance, symptom onset and biomarker changes in autosomal dominant AD (ADAD) and Down syndrome-associated AD (DSAD). In ADAD, there are conflicting findings regarding the role of *APOE*4 in penetrance and symptom onset, with notable research gaps in amyloid and tau changes. However elevated neurofilament light chain (NfL) concentrations have been observed in *PSEN1* and *APOE4* carriers. The figure also highlights limited evidence for sex-*APOE* interactions in symptom onset and amyloid changes in ADAD, with many research gaps (RG). In DSAD, *APOE4* shows an effect on penetrance, symptom onset, and biomarker changes. Regarding sex*, APOE4* appears to influence symptom onset more in females than males, with a stronger impact on amyloid and neurodegeneration, though there are RG in tau biomarker changes.

Second, while the limited research necessitates cautious interpretation, and there are mixed results in the literature, the more recent studies with larger sample sizes show minimal sex differences in disease penetrance, symptom onset and both clinical and biomarker changes in these populations, with some nuances. For example, females in advanced stages of ADAD and *APOE4* homozygotes may show increased neurodegeneration compared to males with similar cognitive outcomes, possibly reflecting greater cognitive reserve. See Figure 2 for an illustration on sex differences in penetrance, symptom onset, and biomarker progression across genetically determined AD populations. Additionally, an interaction between *APOE*4 and sex has been observed in DSAD, aligning with findings in sporadic AD (Figure 3).

Emerging multi-omics studies have revealed sex-specific molecular patterns in ADAD, DSAD and *APOE4* carriers, with a notable absence of research specifically focused on *APOE*4 homozygotes. Transcriptomic analyses in DSAD highlighted pronounced neuroinflammatory and glial gene expression differences in females, while proteomic studies in ADAD showed largely sex-independent biomarker trajectories but raised questions about differential neurodegenerative processes. In *APOE4* carriers, metabolomic analyses revealed distinct vulnerabilities in energy production pathways, particularly among females, with links to increased p-tau levels and reduced brain glucose uptake. These findings contrast with those in sporadic AD, where multi-omics analyses consistently report more widespread sex differences across immune, synaptic, and metabolic pathways. However, it remains unclear whether these differences are truly less pronounced or simply understudied compared to sporadic AD. Integrating transcriptomics, proteomics, and metabolomics is critical to understanding the interplay between sex and genetic risk factors, advancing sex-specific biomarkers and therapeutic strategies and further research is warranted in these genetic populations.

The smaller sex differences observed in genetically determined AD compared to sporadic AD suggest a lesser influence from sex-specific factors in genetically determined AD. Cognitive resilience and brain reserve may offer less protection in genetic AD, where lifestyle and environmental factors, more influential in sporadic AD, could also play a role in observed differences. Additionally, sporadic AD often involves other pathologies, such as vascular dysfunction, that play a limited role in genetically determined AD. These distinctions highlight the importance of tailoring research and interventions to the specific mechanisms and timelines of genetic and sporadic forms of AD while considering the role of sex in disease pathogenesis.

## 6 Priority areas for future research

To comprehensively understand the role of sex in genetically determined AD populations and develop personalized diagnostic, preventive, and therapeutic strategies, future research efforts address the following areas (Table 2):

**Table 2:** Key research priorities for addressing sex in genetically determined Alzheimer’s disease.

Area	Recommendations
Adhere to guidelines on sex-related research	Clearly define hypotheses related to sex differences and power studies to detect these differences. Ensure adequate sample sizes and consistently report demographic characteristics. Stratify analyses for males and females. Adjust for confounders like age, socioeconomic status, comorbid conditions. Promote and use longitudinal designs and incorporate hormonal and genetic data. Apply appropriate statistical models for repeated measures and time-dependent effects.
Communicate results following the MAGIC principles	Magnitude: Accurately describe the extent of any differences, including areas where no differences are found. Accuracy: Carefully interpret variables considering biological, social, and cultural factors. Generalizability: Be cautious about how widely results can be applied. Inflation: Avoid overstating the importance of findings. Credibility: Acknowledge the fit of findings with existing research and the limitations of the study.
Diversity, equity, and inclusion (DEI)	Prioritize research that includes racially and ethnically diverse populations to address disparities in AD risk, progression, and outcomes. Perform stratified analyses to explore how genetic, environmental, and sex-specific factors differ across racial and ethnic groups. Foster international collaborations to expand the inclusion of underrepresented populations, particularly in underdeveloped and underserved regions. Address barriers to participation in AD research, such as accessibility, mistrust, and financial constraints, to ensure equitable representation in study cohorts. Ensure that data collection and analyses consider the cultural and social contexts of participants, enabling more inclusive and accurate findings.
Integration, expansion and comparison of data	Expand the work on consolidating global research networks for collaboration and data sharing in these populations (e.g., ADNI, DIAN, ABC-DS) to other countries. Ensure diverse populations are included by fostering international collaborations and supporting research in underdeveloped countries. Implement standardized protocols for data collection, analysis, reporting, and quality control that are cross-cultural sensitive and specific. Develop a unified database consolidating data from DSAD, ADAD, and *APOE4* homozygote cohorts.
Sex-specific cognitive assessments	Develop and validate cognitive assessment tools tailored to capture early cognitive decline in females. Traditional verbal-dependent memory tests may not detect early declines in females due to their inherent strengths in these areas. Instead, tools that assess cognitive domains where females do not have a preexisting advantage, such as nonverbal problem-solving, or mathematical reasoning, may provide more accurate early detection of cognitive decline.
Address specific needs in DSAD population	Individuals with DS have several comorbid medical conditions. Investigate sex-related differences in the prevalence of conditions like hypothyroidism, late-onset epilepsy or cardiac disorders. Conduct separate analyses for males and females on how specific comorbidities impact penetrance, symptom onset and clinical progression, as well as biomarker progression within the AT(N) framework. This approach will allow us will allow us to conduct research with gender perspective in this population. In addition, because individuals with DS have ID, specific cognitive outcome measures to capture decline should be tailored for this population.

In summary, while current evidence suggests that sex-specific differences in genetically determined AD are small regarding overall disease penetrance and progression, certain variations may exist. These findings highlight the need for further research into both sex and genetic factors in AD outcomes to enhance our understanding and management of the disease in genetically determined populations. Longitudinal studies that are robustly powered and adhere to sex-specific guidelines (Castro-Aldrete et al., 2023) are critical. It is essential that these studies do not merely control for sex as a confounding variable but instead perform stratified analyses by sex. This approach enables a deeper understanding of how sex-specific factors influence key outcomes, such as disease penetrance, symptom onset, and biomarker trajectories, which may otherwise be obscured in aggregate data. Similarly, race-specific analyses should be conducted to elucidate how the interaction of sex and race impacts AD outcomes in genetically determined forms such as DSAD, ADAD, and *APOE44.* In this regard, research efforts should prioritize including underrepresented groups, such as Black, Hispanic, and Asian individuals, to better understand how genetic, environmental, and sex-specific factors interact in diverse populations. Current studies largely focus on non-Hispanic White populations, limiting the generalizability of findings. Addressing this gap is critical for ensuring equitable and comprehensive insights into AD risk and progression across different populations. Studies should also include variables unique to each sex, such as hormonal status, to ensure a comprehensive assessment of their impact on AD outcomes. Hormonal influences, such as those occurring during menopause or andropause, likely interact with genetic predispositions like *APOE4*, influencing disease risk and progression which could be specific to DSAD or ADAD. Cognitive assessment tools should be developed and validated to capture early declines specific to each sex. Current assessments, which often focus on verbal episodic memory, may overlook early declines in females due to their inherent strengths in this domain. Tools that assess cognitive areas where females lack a preexisting advantage, such as visuospatial or executive functions, may provide earlier and more accurate detection of cognitive decline. In this same line, individuals with DS require specialized cognitive tools capable of distinguishing early decline from variations in premorbid intellectual disability. Sex-related differences in cognitive resilience also warrant further study, particularly in DSAD, ADAD, and *APOE44* populations, where females often exhibit preserved verbal memory despite underlying neurodegeneration. Understanding these differences has significant implications for precision medicine strategies aimed at enhancing cognitive resilience in genetically determined AD populations. Finally, research findings should always be communicated clearly and transparently, adhering to the MAGIC principles [Magnitude, Accuracy, Generalizability, Inflation, Credibility (Rippon et al., 2024)]. This ensures that findings are impactful and credible while avoiding overstatement of results or misinterpretation. By addressing these gaps and prioritizing the outlined areas, future research can advance understanding of the role of sex in genetically determined AD populations. This will enable the development of more effective, personalized interventions and therapies to improve outcomes for both males and females.
